# Significant but Overlooked: Atmospheric HONO Formation from Surface Ammonium Oxidation with Superoxide Radicals

**DOI:** 10.34133/research.0819

**Published:** 2025-08-12

**Authors:** Hong Wang, Zehui Hu, Shujun Liu, Xin Zhang, Meijia Jiang, Yanjuan Sun, Fan Dong

**Affiliations:** ^1^ Research Center for Carbon-Neutral Environmental & Energy Technology, Institute of Fundamental and Frontier Sciences, University of Electronic Science and Technology of China, Chengdu 611731, China.; ^2^ School of Resources and Environmental, University of Electronic Science and Technology of China, Chengdu 611731, China.; ^3^ Key Laboratory of Atmospheric Pollution Causes, Prevention and Control in Complex Terrain of Chengdu-Chongqing Region, Ministry of Ecology and Environment, PRC, Chongqing 401147, China.

## Abstract

Resolving the sources of HONO formation is an indispensable aspect in understanding the enhancement of atmospheric oxidation. However, the contributing sources of high HONO formation rate remain unclear during humid haze episodes. The photochemical conversion of surface nitrate (NO_3_^−^), considered as the dominant contributor to the daytime HONO generation, exhibits severe constraint under high relative humidity (RH) conditions. Unexpectedly, ammonium (NH_4_^+^) on the surface of photoactive mineral dust shows a gradual acceleration of HONO generation with increasing RH under simulated solar irradiation, especially at high RH. This reversed observation stems from a change in the photochemical pathway for the HONO formation from NO_3_^−^ and NH_4_^+^. The photochemical conversion of surface NO_3_^−^ is determined by photogenerated electrons (NO_3_^−^→NO_2_→NO_2_^−^→HONO), while the superoxide radical (∙O_2_^−^) generated during photochemical reaction drives the surface NH_4_^+^ to directly form HONO with the pathway (NH_4_^+^∙+∙O_2_^−^→NO_2_^−^ + H_2_O→HONO). Under high RH conditions, oxygen molecules (O_2_) have greatly better access to photogenerated electrons than NO_2_, resulting in an interruption of the procedure from NO_2_ to NO_2_^−^ during NO_3_^−^ conversion. Therefore, the favorably generated ∙O_2_^−^ fuels the photochemical conversion of surface NH_4_^+^ while inhibiting the conversion of NO_3_^−^ to diurnal HONO formation. This work highlights the overlooked contribution of HONO formation from NH_4_^+^, especially under high RH conditions, and advances the understanding of a renewed role for O_2_ in atmospheric chemical processes.

## Introduction

In the tropospheric atmosphere, nitrous acid (HONO) roughly contributes to 30% to 50% of the hydroxyl radical (·OH) production, directly leading to increased atmospheric oxidation and secondary aerosol formation [1–3]. Parsing the sources of HONO becomes a priority in evaluating atmospheric ·OH budgets [4,5]. For daytime HONO levels, the heterogeneous conversion of nitrogen dioxide (NO_2_) and photolysis of nitrate (NO_3_^−^) are considered significant contributing pathways, which may also be enhanced on diverse surfaces in the atmosphere, such as sea salt aerosol, soot, mineral dust, and architectural surfaces [6–10]. Notably, it is found that the low reaction rates of HONO formation by dimerization of NO_2_ on many environmental surfaces result in a poor explanation for observations [11–13]. Ma et al. [14] reported that the photolysis of NO_3_^−^ on the surface of typical photoactive mineral dust, TiO_2_, has a prominent contribution to HONO generation. However, water displays an inhibitory effect on HONO formation from the surface NO_3_^−^ photolysis under high relative humidity (RH) conditions [14]. This is a paradox that high HONO formation rates are determined during humid haze episodes [12,15]. Hence, there should be unknown sources for the daytime HONO budget, particularly in high RH conditions.

Ammonia (NH_3_) is an abundant reduced nitrogen species in the atmosphere [16–20]. NH_3_ exhibits increasing emissions over the past decades, even up to parts per million (ppmv) levels in highly polluted regions, and has an important effect on the atmospheric reaction [13,21–23]. NH_3_ is effective in promoting the hydrolysis reaction of NO_2_ to form HONO by theoretical calculation because the energy barrier of NO_2_ conversion is lowered dramatically [24,25]. A new pathway has been discovered, where NH_3_ assists in the conversion of NO_2_ into HONO under ultraviolet light irradiation on urban surfaces, in which NH_3_ acts as a hydrogen carrier to facilitate the transfer of hydrogen from H_2_O to monomeric NO_2_ [12]. In addition, Kebede et al. [26] reported that HONO can be formed directly by NH_3_ photooxidation on the TiO_2_ surface. This suggests a potential contributing role of NH_3_ to diurnal HONO formation. Regrettably, the conversion of NH_3_ to HONO remained unsatisfactory under high RH conditions. When the RH was increased from 40% to 95%, HONO formation was greatly inhibited, with a decrease in the generation amount of about 86% [26]. This likely stems from the NH₃ conversion process being governed by its collision efficiency with the surface, while the competitive occupation of reactive sites by water molecules may significantly hinder the reaction under high RH conditions [27]. Notably, in the atmosphere, NH_3_ prefers to form particulate ammonium (NH_4_^+^) through gas-to-particle conversion, which is further augmented in pollution episodes [28]. As the NH_3_ emissions increase, so does the share of NH_4_^+^ in the atmospheric environment. Moreover, NH_4_^+^ often combines with NO_3_^−^ in the form of NH_4_NO_3_ on the particulate surface, particularly in ammonia-rich areas [29,30]. Accordingly, there is a high incidence of NH_4_^+^ being triggered by photoactive particulates to undergo photochemical conversion. More importantly, in contrast to NH_3_, NH_4_^+^ adsorbed directly on the surface of particulate matter has the potential to diminish the occupation of reaction sites by water molecules. Hence, it is worth exploring whether the NH_4_^+^ on mineral dust can contribute to HONO formation under high RH conditions.

Herein, the photochemical conversion from NH_4_^+^ to HONO on the surface of the typical photoactive mineral dust under a variety of RH conditions was discovered and validated as a new pathway. NH_4_NO_3_, which is the most common ammonium salt in the atmosphere, was chosen as a proxy. Meanwhile, NaNO_3,_ as a representative of atmospheric nitrate salts, was used as a comparison to elucidate the discrepancy between NH_4_^+^ and NO_3_^−^ contributing to HONO formation. The dynamic evolution of surface NH_4_^+^ and NO_3_^−^ was scrutinized by in situ diffuse reflectance Fourier transform infrared spectroscopy (DRIFTS). The trapping experiments of active species were combined to resolve the pathways of HONO formation. Under high RH conditions, the role played by oxygen molecules (O_2_) was recognized during the photochemical conversion of NH_4_^+^ and NO_3_^−^ through conditioned experiments, ^15^N-isotope labeling, and in situ electron paramagnetic resonance (EPR) spectroscopy. This study reveals an overlooked but significant pathway by which NH_4_^+^ can contribute to atmospheric HONO formation, especially under high RH conditions.

## Results and Discussion

### HONO production from the photochemical conversion of ammonium

**Fig. 1.**
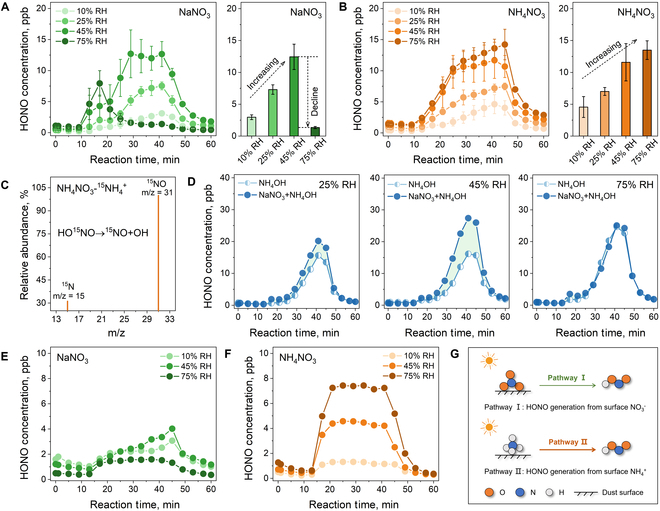
HONO generation evaluation for photochemical conversion of surface NO_3_^−^ and NH_4_^+^ at different RH conditions: HONO formation from NaNO_3_ (A, left) and NH_4_NO_3_ (B, left) adsorbed on TiO_2_ surface with time of illumination at different RH conditions. The histograms represent the concentrations of HONO formation from NaNO_3_ (A, right) and NH_4_NO_3_ (B, right) at 30 min of illumination. Gas chromatography–mass spectrometry spectra of the HONO decomposition product (NO) from the photochemical conversion of NH_4_NO_3_-^15^NH_4_^+^ at high RH conditions (C). HONO formation from NH_4_OH and NaNO_3_ + NH_4_OH mixture adsorbed on TiO_2_ surface under simulated solar irradiation at 25% RH, 45% RH, and 75% RH conditions (D). HONO formation from NaNO_3_ (E) and NH_4_NO_3_ (F) adsorbed on the A1 dust surface under simulated solar irradiation at different RH conditions. (G) Diagram of the HONO formation pathway from surface NO_3_^−^ and NH_4_^+^.

Figure 1A and B displays the time-dependent production of HONO when NaNO_3_ and NH_4_NO_3_ are presented on the surface of typical photoactive mineral dust, TiO_2_, at 10%, 25%, 45%, and 75% RH. To clearly distinguish the variation trend of HONO production with the increased RH, histograms of product concentrations are also shown in Fig. 1A (right) and B (right). All samples released HONO upon simulated solar light irradiation, and the generated concentration gradually increased when the RH was adjusted from 10% to 45%. This fact owes to the important roles of water molecules in providing hydrogen protons and promoting decomposition [31,32]. However, at a high RH level of 75%, the HONO production from the photochemical conversion of surface NaNO_3_ sharply declined. This decrease phenomenon was previously attributed to the deliquescence of NaNO_3_ at 75% RH [14,33]. However, as for NH_4_NO_3_, its deliquescence point is 62% RH [34,35]. The trend of gradual increase in HONO generation was still maintained for surface NH_4_NO_3_ when the RH reached 75% (Fig. 1B). These results suggest that there should be an unidentified mechanism dominating the HONO production during the photochemical conversion of surface NH_4_NO_3_.

Compared with NaNO_3_, which contains NO_3_^−^ that results in the production of HONO, NH_4_NO_3_ possesses other sources of nitrogen, i.e., NH_4_^+^, which may contribute to the release of HONO. The experiment using isotope-labeled NH_4_NO_3_-^15^NH_4_^+^ was conducted under high RH conditions (Fig. 1C). The mass spectra of the HONO decomposition product (NO) are similar to that of the standard ^15^NO, indicating that the nitrogen element in the HONO product can derive from NH_4_^+^. Subsequently, NH_4_OH was used as a single representative of NH_4_^+^ to load on the TiO_2_ surface for photochemical reaction testing. As shown in Fig. 1D, appreciable HONO concentrations were observed during the irradiation at 25%, 45%, and 75% RH. This indicates that the single NH_4_^+^ itself is available as a new source of daytime HONO.

Furthermore, samples loading both NH_4_OH and NaNO_3_ were investigated to understand the contribution of NH_4_^+^ and NO_3_^−^ to HONO formation at different RH. As shown in the green shaded area of Fig. 1D, there is an additional HONO concentration in comparison to the pure NH_4_OH sample. Moreover, the green shaded area was enlarged as the RH increased from 25% to 45% (Fig. 1D), which is consistent with the phenomenon in Fig. 1A that the photochemical conversion of surface NO_3_^−^ to HONO can be facilitated by water molecules in the moderate RH range. Notably, at a high RH level of 75%, there was hardly a green shaded area. Pure NH_4_OH and NH_4_OH/NaNO_3_ mixture samples generated nearly identical HONO concentrations under irradiation. These results consolidate that, under high RH conditions, the photochemical conversion of surface NO_3_^−^ may be a relatively weak contribution to HONO production in the atmosphere. Conversely, NH_4_^+^ is probably the vital benefactor to the enhanced atmospheric oxidation capacity during haze pollution, especially under high RH conditions.

To verify the generalization of the above results to the atmospheric environment, the photochemical conversion of NaNO_3_ and NH_4_NO_3_ was further carried out on the surface of authentic mineral dust (A1 dust) under simulated solar light. As shown in Fig. 1E and F, as the RH changes from 45% to 75%, the HONO formation presented a declining tendency for the photochemical conversion of NaNO_3_, while an increasing trend was still maintained for that of NH_4_NO_3_. Therefore, abundant NH_4_^+^, which is commonly coexisting with NO_3_^−^ on the surface of photoactive mineral dust, may be a seriously overlooked source of daytime HONO in the atmospheric environment, especially under high RH conditions. As shown in Fig. 1G, in addition to the photochemical conversion of surface NO_3_^−^ (pathway I), there should be a new pathway of surface NH_4_^+^ contributing to daytime HONO formation in the atmosphere (pathway II).

### HONO formation mechanisms for photochemical conversion of ammonium

To understand the HONO generation pathways, in situ DRIFTS was employed to investigate the dynamic evolution of surface NO_3_^−^ and NH_4_^+^ under light irradiation at low (10%) and high (75%) RH. For the surface NaNO_3_ sample under low RH conditions (Fig. S1), once the light was started, the observed variation in infrared spectra was the NO_2_ (1,626 cm^−1^), NO_2_^−^ (1,426 cm^−1^), and NO_3_^−^ (1,578, 1,570, 1,296, and 1,259 cm^−1^) bands [32,36–38]. Among them, NO_2_ and NO_2_^−^ belong to the products of photochemical NO_3_^−^ conversion, while the generated NO_3_^−^ is due to the re-oxidation of photochemical products by active species, such as h^+^, ·O_2_^−^, and ·OH [39,40]. The peak intensity is enhanced progressively with increasing light duration. The accumulation of NO_3_^−^ is particularly prominent. This suggests that most of the photochemical products are forced to be re-oxidized to NO_3_^−^ at low RH, due to the shortage of water molecules on the surface. Similar products and evolution were found during the photochemical conversion of NH_4_OH (Fig. S2) and NH_4_NO_3_ (Fig. S3) under low RH conditions. Therefore, it is inferred that the photochemical conversion of both NO_3_^−^ and NH_4_^+^ requires water molecules to provide adequate hydrogen protons for the hydrogenation reactions to achieve HONO formation.

**Fig. 2.**
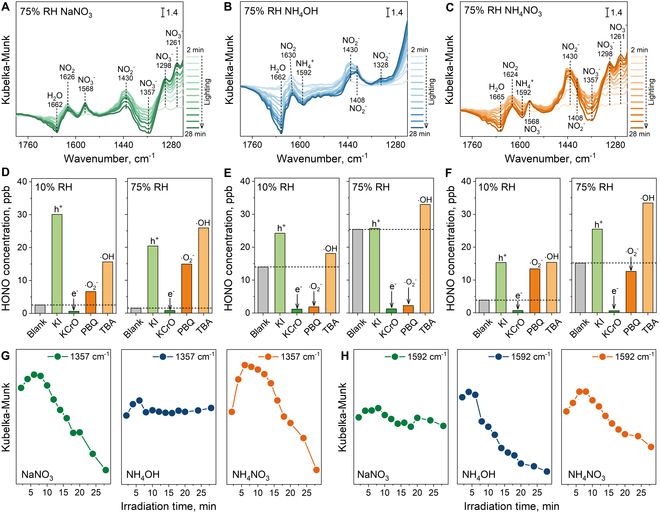
Dynamic evolution and radical trapping for surface NO_3_^−^ and NH_4_^+^: In situ DRIFTS of NaNO_3_ (A), NH_4_OH (B), and NH_4_NO_3_ (C) adsorbed on the TiO_2_ surface under simulated solar irradiation at 75% RH. The variation of HONO-generating concentrations of NaNO_3_ at 10% (D, left) RH and 75% RH (D, right), NH_4_OH at 10% (E, left) RH and 75% RH (E, right), and NH_4_NO_3_ at 10% (F, left) RH and 75% RH (F, right) with trapping experiments of active species. The detailed evolution of the NO_3_^−^ peak with time at 1,357 cm^−1^ (G) and NH_4_^+^ peak with time at 1,592 cm^−1^ (H) in infrared spectra of NaNO_3_, NH_4_OH, and NH_4_NO_3_.

At high RH conditions, the photochemical products of the samples are consistent with those under low RH (Fig. 2A to C). Differently, as shown in Fig. 2A, the depletion of the surface NO_3_^−^ band at 1,357 cm^−1^ and the generation of NO_2_^−^ band at 1,430 cm^−1^ became evident during the photochemical NO_3_^−^ conversion. The peak intensity of generated NO_3_^−^ (1,568, 1,298, and 1,261 cm^−1^) also moderated. These facts suggest that adequate water molecules advance the surface NO_3_^−^ conversion and slow down the re-oxidation of photochemical products. Generally, the photochemical conversion occurring on the surface of photoactive particulate matter is primarily driven by the generated active species [39,41]. Under light irradiation, photogenerated electrons and holes are derived from photoexcitation of photoactive particulate matter. Then, the generated electrons reduce O_2_ to ·O_2_^−^ and holes oxidize H_2_O to ·OH. As shown in Figs. S4 to S6, the photogenerated carriers, ·O_2_^−^, and ·OH were detected in the TiO_2_ sample under light conditions. To further confirm the key active species that drive the photochemical conversion of surface NO_3_^−^, we performed corresponding capture experiments. As shown in Fig. 2D, under both humidity conditions, there was a significant inhibition of HONO production only after the photogenerated electrons were captured, demonstrating that photogenerated electrons are the crucial driver of the photochemical NO_3_^−^ conversion. Coupled with the detected intermediates in Fig. 2A and their valence state of nitrogen element, the photochemical conversion pathway from NO_3_^−^ to HONO is reasoned to be NO_3_^−^→NO_2_→NO_2_^−^→HONO, as shown in Eqs. 1 to 3. The other active species serve primarily to re-oxidize the photochemical products to NO_3_^−^ (Eq. 4) so that, after trapping them, more HONO can be monitored. Besides, the accumulation of NO_3_^−^ can be observed in the infrared spectrum (Fig. 2A).

$${{\mathrm{NO}}_{3}}^{-}+e^{-}\to {\mathrm{NO}}_{2}+O^{-}$$ (1)

$${\mathrm{NO}}_{2}+e^{-}\to {{\mathrm{NO}}_{2}}^{-}$$ (2)

$${{\mathrm{NO}}_{2}}^{-}+H_{2}O\to \text{HONO}+{\mathrm{OH}}^{-}$$ (3)

$${\mathrm{NO}}_{2}/{{\mathrm{NO}}_{2}}^{-}+h^{+}/\cdot {O_{2}}^{-}/\mathrm{OH}\to {{\mathrm{NO}}_{3}}^{-}$$ (4)

As for the surface NH_4_OH sample under high RH conditions (Fig. 2B), with increasing light time, the characteristic peak belonging to NH_4_^+^ at 1,592 cm^−1^ was consumed significantly [42]. Meanwhile, the peaks of NO_2_ at 1,630 cm^−1^ and NO_2_^−^ at 1,430, 1,408, and 1,328 cm^−1^ emerged, suggesting that NH_4_^+^ mainly undergoes a photooxidation process. Notably, the conversion of NH_4_^+^ to HONO was greatly hindered when either photogenerated electrons or ·O_2_^−^ was captured (Fig. 2E). Given that the generation of ·O_2_^−^ also requires the presence of photogenerated electrons (Eq. 5), the role of photogenerated electrons and ·O_2_^−^ for the photochemical conversion of NH_4_^+^ was further identified. The NH_4_OH sample was subjected to continuous-flow daylight tests in an argon (oxygen-free) atmosphere at 75% RH. As shown in Fig. S7, the HONO formation became almost negligible. This result demonstrated that, for the photochemical conversion of NH_4_^+^ to HONO, the oxygen (O_2_) is the essential driver, which acquires photogenerated electrons to form ·O_2_^−^ and then gradually oxidizes NH_4_^+^ to NO_2_^−^. It is unfortunate that more transient intermediates were not monitored, possibly due to the rapidity of the reaction or the overlap of the spectral region [26,43]. According to the generation of NO_2_^−^ in Fig. 2B, the total reaction for the conversion of NH_4_^+^ to NO_2_^−^ driven by ·O_2_^−^ is shown in Eq. 6. Significantly, there is a great difference in the photochemical conversion pathways of NH_4_^+^ and NO_3_^−^. The photochemical pathway of NH_4_^+^ is NH_4_^+^→NO_2_^−^→HONO. ·O_2_^−^ is used for the NH_4_^+^ conversion, and adequate water molecules available for NO_2_^−^ to HONO formation mitigate the re-oxidation of photochemical products, so the accumulated NO_3_^−^ peaks hardly appeared in Fig. 2B. Besides,

$$O_{2}+e^{-}\to \cdot {O_{2}}^{-}$$ (5)

$${{\mathrm{NH}}_{4}}^{+}+2\cdot {O_{2}}^{-}\to {{\mathrm{NO}}_{2}}^{-}+{\text{2H}}_{2}O$$ (6)

For the surface NH_4_NO_3_ sample, its infrared spectra exhibited photochemical conversion characteristics of both species, NO_3_^−^ and NH_4_^+^, under high humidity conditions (Fig. 2C). Detailly, as shown in Fig. 2G and H, the depletion that is unique to NO_3_^−^ (1,357 cm^−1^) in the surface NaNO_3_ sample and NH_4_^+^ (1,592 cm^−1^) in the surface NH_4_OH sample was observed in the surface NH_4_NO_3_ sample. This indicates that the photochemical conversion processes of NO_3_^−^ and NH_4_^+^ are occurring simultaneously in the surface NH_4_NO_3_ sample. Correspondingly, when photogenerated electrons were quenched, the HONO formation was significantly suppressed (Fig. 2F). When ·O_2_^−^ was quenched, surface NH_4_NO_3_ allowed decent HONO formation at low RH conditions. This is because, although the photochemical conversion of NH_4_^+^ struggles, some NO_3_^−^ photochemical products also escape ·O_2_^−^ re-oxidization to participate in HONO generation. Notably, under high RH, photochemical conversion from NH_4_NO_3_ to HONO displayed depression after ·O_2_^−^ quenching, reaffirming that the surface NO_3_^−^ conversion is restricted and surface NH_4_^+^ becomes the main contributing source of daytime HONO level.

### Effect of O_2_ on the photochemical conversion from ammonium to HONO

**Fig. 3.**
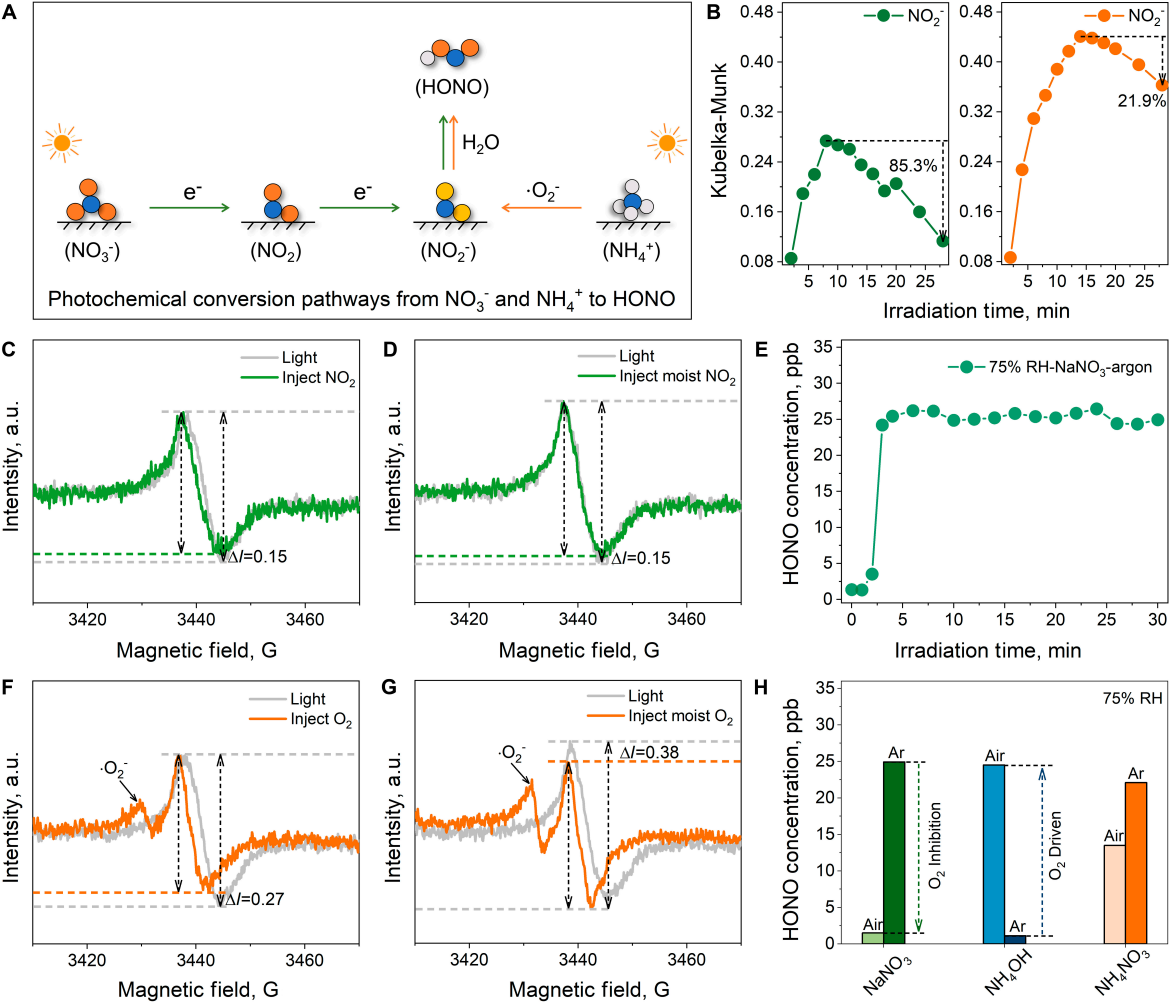
Reaction mechanism for photochemical conversion of surface NH_4_^+^ driven by ·O_2_^−^. (A) Diagram of photochemical conversion pathways from surface NO_3_^−^ and NH_4_^+^ to HONO. (B) Detailed evolution of the NO_2_^−^ peak in infrared spectra of NaNO_3_ and NH_4_NO_3_. In situ EPR of NO_2_ (C) and moist NO_2_ (D). HONO formation from NaNO_3_ and NH_4_NO_3_ adsorbed on TiO_2_ surface under simulated solar irradiation at 75% RH conditions in argon atmosphere (E). In situ EPR of O_2_ (F) and moist O_2_ (G). HONO formation from NaNO_3_, NH_4_OH, and NH_4_NO_3_ adsorbed on TiO_2_ surface under simulated solar irradiation at 75% RH conditions in air and argon atmosphere (H).

To sum up, as shown in Fig. 3A, the photochemical conversion of surface NH_4_^+^ is mainly driven by ·O_2_^−^, whereas that of surface NO_3_^−^ relies on photogenerated electrons. Furthermore, the dynamic evolution of the critical species (NO_2_^−^) during the NH_4_^+^ and NO_3_^−^ conversion was dissected to elucidate the cause of their difference in HONO production under high RH conditions. As shown in Fig. 3B (left), although NO_3_^−^ was able to generate a certain amount of NO_2_^−^ in the early stage, the surface NO_2_^−^ was rapidly reduced by 85.3% to near depletion as the light reaction proceeded. Differently, the accumulation of NO_2_^−^ during the conversion of surface NH_4_NO_3_ nearly doubled, and a favorable production–consumption equilibrium was maintained, with a declining amount of only 21.9% as the reaction progressed (Fig. 3B, right). Accordingly, when adequate water molecules are present, NH_4_NO_3_ can induce the formation of enough NO_2_^−^ for HONO production, whereas NaNO_3_ cannot. Curiously, the depletion of surface NO_3_^−^ was occurring as usual during photochemical conversion (Fig. 2G). The other reactive nitrogen species were monitored when surface NaNO_3_ was irradiated at 75% RH. As shown in Fig. S8, unlike the case of HONO production, surface NaNO_3_ generated appreciable amounts of NO_2_ under high RH, suggesting that the conversion from NO_3_^−^ to NO_2_ is smooth. Hence, it can be inferred that the inability of NO_3_^−^ to HONO is mainly derived from the hindered NO_2_^−^ generation at the step of NO_2_ gaining electrons (NO_2_ + e^−^→NO_2_^−^).

During photochemical reactions, for NO_2_, O_2_ may be a formidable competitor for expending photogenerated electrons, as it tends to be activated to form ·O_2_^−^. In situ EPR was applied to investigate whether O_2_ has a superior ability to acquire photogenerated electrons than NO_2_ on the surface [44]. Under exposure to light, the TiO_2_ sample produces abundant photogenerated electrons, so a greatly enhanced EPR signal intensity emerged (Fig. S9). Subsequently, the NO_2_ atmosphere was introduced into the in situ reaction chamber. A slight decrease in EPR signal intensity (Δ = 0.15) can be seen in Fig. 3C, indicating that a part of the photogenerated electrons has been consumed by NO_2_. When water molecules were present in the introduced NO_2_ atmosphere, the consumption of photogeneration electrons by NO_2_ was not promoted, and the decrease in the EPR signal intensity remained at 0.15 (Fig. 3D). Prominently, when the O_2_ atmosphere was introduced, the intensity of the EPR signal drop reached 0.27 (Fig. 3F). This result proves that O_2_ indeed has a greater ability to acquire photogenerated electrons than NO_2_. Meanwhile, the EPR signal belonging to ·O_2_^−^ appeared, indicating that O_2_ is activated to form ·O_2_^−^ after capturing the photogenerated electron [45]. The O_2_ atmosphere accompanying the water molecules was also investigated. Surprisingly, as shown in Fig. 3J, the more significant photogenerated electron consumption appeared (Δ = 0.38), along with a stronger EPR signal belonging to ·O_2_^−^. This result manifests that water molecules can greatly enhance the acquisition of photogenerated electrons by O_2_. Accordingly, during photochemical reactions, O_2_ enables the consumption of photogenerated electrons in preference to NO_2_, which is further exacerbated under high RH conditions. This variation may result in the inability of NO_2_ to efficiently obtain photogenerated electrons for the conversion to NO_2_^−^ under high RH conditions, thereby interrupting the HONO formation from surface NO_3_^−^.

To identify the inhibitory effect of O_2_ on the photochemical conversion of NO_3_^−^, the daylight tests of samples were placed in an argon atmosphere at 75% RH. As shown in Fig. 3E, decent HONO production appeared. These results confirmed that, under high RH, O_2_ significantly inhibits the acquisition of photogenerated electrons by NO_2_, resulting in the inability of NO_2_^−^ production, and then restricts photochemical conversion from NO_3_^−^ to HONO. Besides, the generated ·O_2_^−^ will re-oxidize the photochemical products to NO_3_^−^, which also retards the production of HONO. However, the NH_4_^+^ pathway requires O_2_ to acquire photogenerated electrons. The generated ·O_2_^−^ is the indispensable driver for HONO formation during the photochemical conversion of NH_4_^+^. Therefore, as shown in Fig. 3H, O_2_ inhibits the conversion of NO_3_^−^ and drives the contribution of NH_4_^+^ to HONO formation under high humidity conditions. The great contribution of NH_4_NO_3_ to HONO formation under air atmosphere mainly derives from the photochemical conversion of surface NH_4_^+^, whereas that under argon atmosphere stems from surface NO_3_^−^.

### Universality of the photochemical conversion from ammonium to HONO driven by ·O_2_^−^

**Fig. 4.**
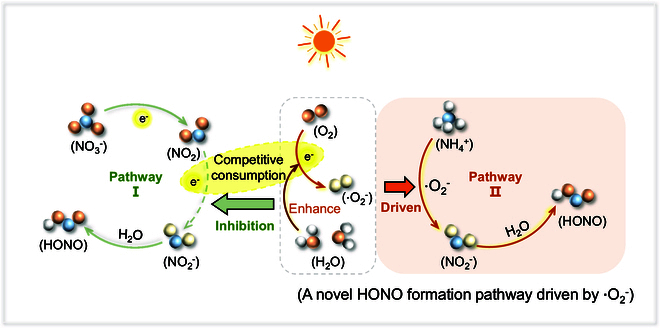
The schematic illustration for the opposite roles of O_2_ during photochemical conversion from NO_3_^−^ and NH_4_^+^ to HONO.

As a result, daytime HONO formation pathways from surface NO_3_^−^ and NH_4_^+^ are shown in Fig. 4. In pathway I, photogenerated electrons are generated on the surface of photoactive mineral dust under solar irradiation, which can trigger the step-by-step conversion of surface NO_3_^−^ to produce HONO (NO_3_^−^→NO_2_→NO_2_^−^→HONO). In the air atmosphere, O_2_ competitively consumes photogenerated electrons to hinder photochemical conversion from surface NO_3_^−^ to HONO, especially under high RH conditions. Differently, in pathway II, ·O_2_^−^ drives the photochemical conversion of NH_4_^+^ to produce HONO (NH_4_^+^ +·O_2_^−^→NO_2_^−^ + H_2_O→HONO), where photogenerated electrons are mainly responsible for the reduction of O_2_ to form ·O_2_^−^. This is distinct from the previously reported pathway for the photo-oxidation of NH_3_ to generate HONO, which is derived from the reduction of NO_2_, where NO_2_ is the product of the water-catalyzed oxidation of NH_3_ (NH_3_→NO_2_→HONO) [26]. Significantly, NH_4_^+^ has a superior contribution to daytime HONO formation under high RH conditions, because the ·O_2_^−^ generation is promoted by water molecules.

To demonstrate that the photochemical conversion from NH_4_^+^ to HONO was extensive in the atmosphere, the HONO generation from surface (NH_4_)_2_SO_4_ and NH_4_Cl, the proxy for atmospheric ammonium salts, was investigated. As shown in Fig. S10, under solar light irradiation, (NH_4_)_2_SO_4_ and NH_4_Cl also undergo photochemical conversion to produce HONO at high RH conditions. Particularly, when ·O_2_^−^ was trapped during the photochemical conversion of surface (NH_4_)_2_SO_4_ and NH_4_Cl, their HONO generation was significantly inhibited, suggesting that the ·O_2_^−^-driven photochemical conversion from surface NH_4_^+^ to HONO was universal at high RH in the atmosphere. This finding aligns with field observations during humid haze episodes in China, where elevated HONO levels often coincide with high RH and substantial particulate ammonium concentrations [46,47]. Therefore, the proposed mechanism involving photochemical conversion of surface NH_4_^+^ may provide a supplementary explanation for these observations and should be considered in future atmospheric models. Furthermore, for strategies to control NH_4_^+^ and HONO in regions with high ammonia emissions, in addition to reducing ammonia emissions to minimize NH_4_^+^ formation, inhibition of aerosol-phase photochemical reactions may be effective in suppressing the generation of interfacial HONO, e.g., by limiting the ambient RH or passivating reactive surfaces to reduce the generation of ·O_2_^−^. It is worth noting that the actual atmospheric conditions involve more complexity. Further work is needed to assess the effects of conditions, including aerosol phase and acidity, on HONO formation to develop control strategies.

## Conclusion

A significant but overlooked pathway for the daytime HONO formation from the photochemical conversion of NH_4_^+^ is validated on the surface of atmospheric photoactive mineral dust. Significantly, under high RH conditions, the contribution of surface NH_4_^+^ to HONO is prominent when that of surface NO_3_^−^ to HONO is substantially suppressed. This discovery could account for the high HONO formation rate during humid haze episodes. The difference in the photochemical HONO formation pathways from surface NH_4_^+^ and NO_3_^−^ is revealed by in situ DRIFTS and capture experiments. The photochemical conversion of surface NH_4_^+^ is mainly driven by the generation of ·O_2_^−^ after the acquisition of photogenerated electrons by O_2_, while NO_3_^−^ directly relies on the gradual reduction of photogenerated electrons. Furthermore, the results of controlled experiments and in situ EPR clarify the opposite roles of O_2_ during the HONO formation from NH_4_^+^ and NO_3_^−^ under high RH conditions. O_2_ excels at capturing electrons to generate ·O_2_^−^, which can be further enhanced by water molecules, advancing the elevated contribution of NH_4_^+^ to HONO formation. However, for photochemical conversion of surface NO_3_^−^ under high humidity conditions, the step of NO_2_ acquiring photogenerated electrons is highly hindered by the competitive consumption of O_2_ to interrupt the path from NO_3_^−^ to HONO. Besides, the ·O_2_^−^ generated during the photochemical conversion of NO_3_^−^ will re-oxidize its photochemical products and inhibit the HONO formation. This overlooked and universal pathway from NH_4_^+^ to HONO would greatly contribute to the enhancement of atmospheric oxidation under high humidity conditions, which sheds light on the significant role of O_2_ in atmospheric HONO formation.

## Materials and Methods

### Materials

All chemical reagents were of analytical grade and were used in this study without further purification. The commercial TiO_2_ (typical photoactive components of mineral dust), sodium nitrate (NaNO_3_), ammonium nitrate (NH_4_NO_3_), and ammonia (NH_4_OH) were obtained from Sigma-Aldrich. Authentic mineral dust (A1 dust) stems from Arizona Test Dust, the detailed components of which are in the Supplementary Materials.

### Gas-phase product detection

The gas-phase product concentration during the photochemical conversion was monitored in real time by using a HONO analyzer (HONO-WLPAP) and a NOx analyzer (Thermo, model 42i-TL). The sample with 0.10 g of mineral dust and 0.001 g of nitrate or ammonium was homogeneously dried in Petri dishes. The reaction sample was placed in a rectangular reactor sealed with quartz glass. When clean air or argon gas at a flow rate of 1.5 l/min clears the atmosphere in the reaction chamber, the tungsten halogen lamp was used as simulated solar light to trigger the photochemical reaction. The RH in the photochemical reaction was regulated by a humidity generator (FD-HG). The trapping experiments were performed when the trapping agent of the active species was added to the sample. Photogenerated hole trapping, photogenerated electron trapping, superoxide radical (·O_2_^−^) trapping, and hydroxyl radical (·OH) trapping were achieved by adding potassium iodide (KI), potassium dichromate (K_2_Cr_2_O_7_, short for KCrO), p-benzoquinone (PBQ), and tert-butanol (TBA), respectively.

### Photochemical process characterization

The active species, including photogenerated carriers (electrons and holes), ·O_2_^−^, and ·OH, were detected by spin-trapped EPR (Bruker EMX nano) spectrometer using 2,2,6,6-tetramethylpiperidine-1-oxyl (TEMPO) and 5,5-dimethyl-1-pyrroline N-oxide (DMPO). The interaction between the photogenerated electron and the reactant molecules was measured by the in situ EPR spectrometer. The photochemical evolution of nitrate (NO_3_^−^) or ammonium (NH_4_^+^) on the surface of photoactive mineral dust was monitored using in situ diffuse reflection infrared Fourier transform spectrum (in situ DRIFTS, Bruker INVENIO-R). The detailed description of in situ EPR and in situ DRIFTS was provided in the Supplementary Materials.

## Data Availability

All data needed for this study are available in the article and its Supplementary Materials.

## Supplementary Materials

Supplementary 1 Experimental Description Figs. S1 to S10
