# Supplementary material for: Significant but Overlooked: Atmospheric HONO Formation from Surface Ammonium Oxidation with Superoxide Radicals
Source: Research (Wash D C). 2025 Aug 12;8:0819. doi: 10.34133/research.0819 (PMC12340223; doi:10.34133/research.0819)
Supplement: Supplementary 1 — Experimental Description Figs. S1 to S10 [file research.0819.f1.docx]

Supplementary Materials for

**Significant but Overlooked: Atmospheric HONO Formation from Surface Ammonium Oxidation with Superoxide Radicals**

**Hong Wang^1,2,3^, Zehui Hu^1^, Shujun Liu^1^, Xin Zhang^1^, Meijia Jiang^1^, Yanjuan Sun^2,3^, Fan Dong^1,3*^**

^1^ Research Center for Carbon-Neutral Environmental & Energy Technology, Institute of Fundamental and Frontier Sciences, University of Electronic Science and Technology of China, Chengdu 611731, China.

^2^ School of Resources and Environmental, University of Electronic Science and Technology of China, Chengdu 611731, China.

^3^ Key Laboratory of Atmospheric Pollution Causes, Prevention and Control in Complex Terrain of Chengdu-Chongqing Region, Ministry of Ecology and Environment, PRC, Chongqing 401147, China.

* Corresponding author: Fan Dong (dongfan@uestc.edu.cn; dfctbu@126.com)

**This file includes:**

Experimental Description

Figs. S1 to S10

**In Situ DRIFTS Measurement:**

In situ DRIFTS measurements were performed using a Fourier Transform Infrared spectrometer (BRUKER 70v) equipped with a reaction chamber and an MCT detector. The sample was pretreated under Ar flow for 30 min. Then, zero air and irradiation were introduced to initiate surface reactions. Spectra were collected at a resolution of 4 cm^-1^ with 64 scans per spectrum. The dynamic evolution of surface species was repeatedly recorded at 2 min intervals.

**In Situ EPR Measurement:**

The prepared sample is filled into an in situ reaction chamber with a gas system (O_2_, NO_2_, H_2_O vapor, or Argon) and a light source. Gas was introduced into the reaction chamber and through the solid sample. The light was turned on to trigger the photochemical reaction. Meanwhile, the evolution of EPR spectra is recorded on the Bruker EMX nano spectrometer at room temperature.

**Components of A1 dust:**

A1 dust (supplied by Arizona Test Dust) was used as a commercial proxy of authentic mineral dust. The primary components of A1 dust include the SiO_2_ (69.0 ~ 77.0%), Al_2_O_3_ (8.0 ~ 14.0%), Fe_2_O_3_ (4.0 ~ 7.0%), CaO (2.5 ~ 5.5%), K_2_O (2.0 ~ 5.0%), Na_2_O (1.0 ~ 4.0%), MgO (1.0 ~ 2.0%), and TiO_2_ (0.5 ~ 1.0%).


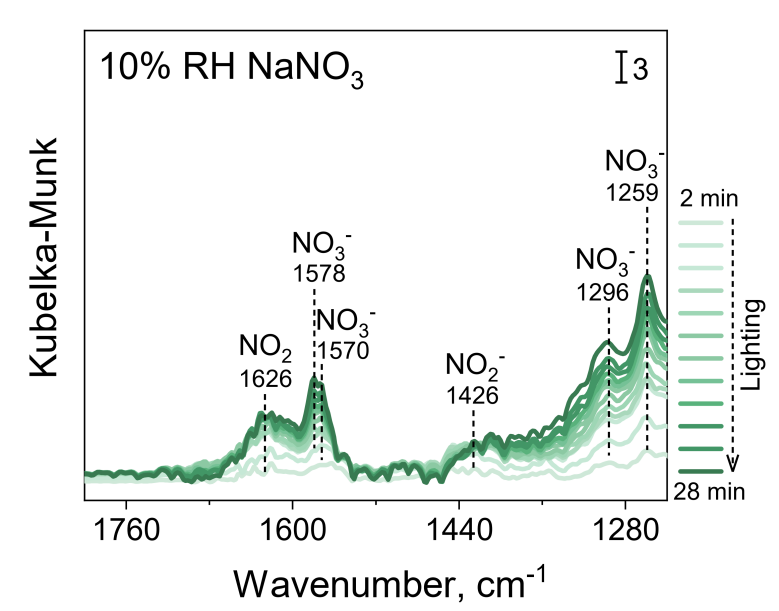


Fig. S1. In situ DRIFTS of NaNO_3_ adsorbed on TiO_2_ surface under simulated solar irradiation at 10% RH.


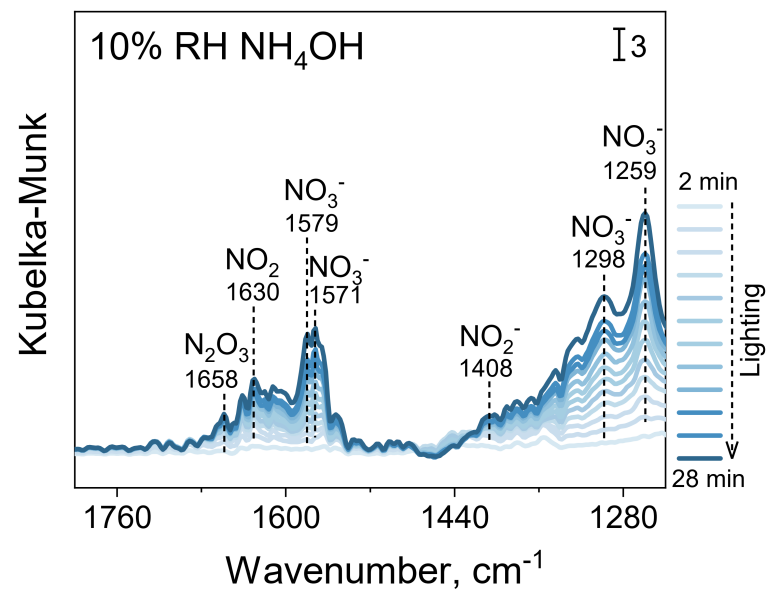


Fig. S2. In situ DRIFTS of NH_4_OH adsorbed on TiO_2_ surface under simulated solar irradiation at 10% RH.


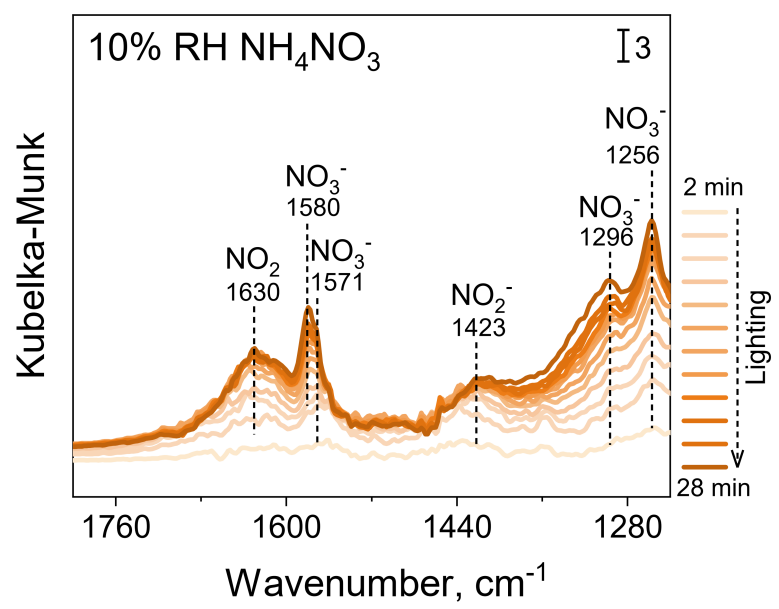


Fig. S3. In situ DRIFTS of NH_4_NO_3_ adsorbed on TiO_2_ surface under simulated solar irradiation at 10% RH.


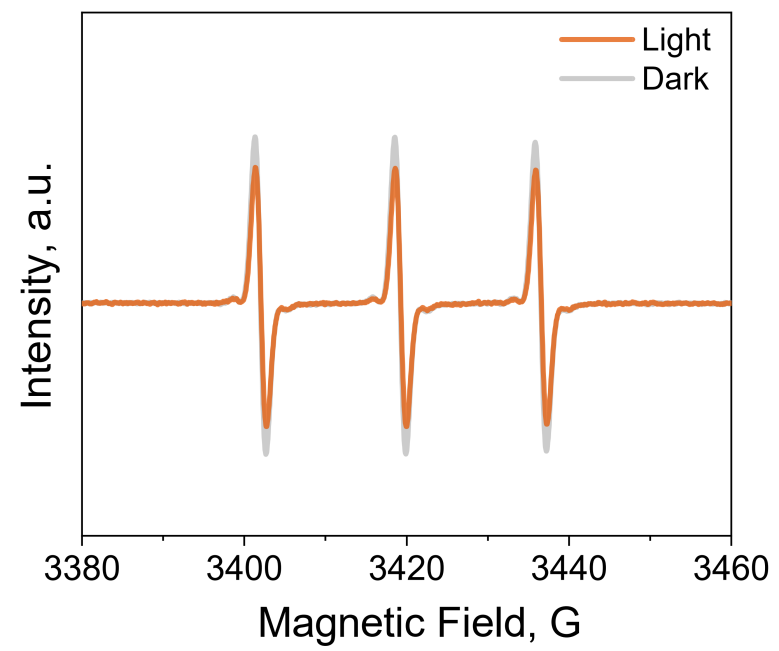


Fig. S4. EPR spectra of photogenerated carriers (electrons and holes).


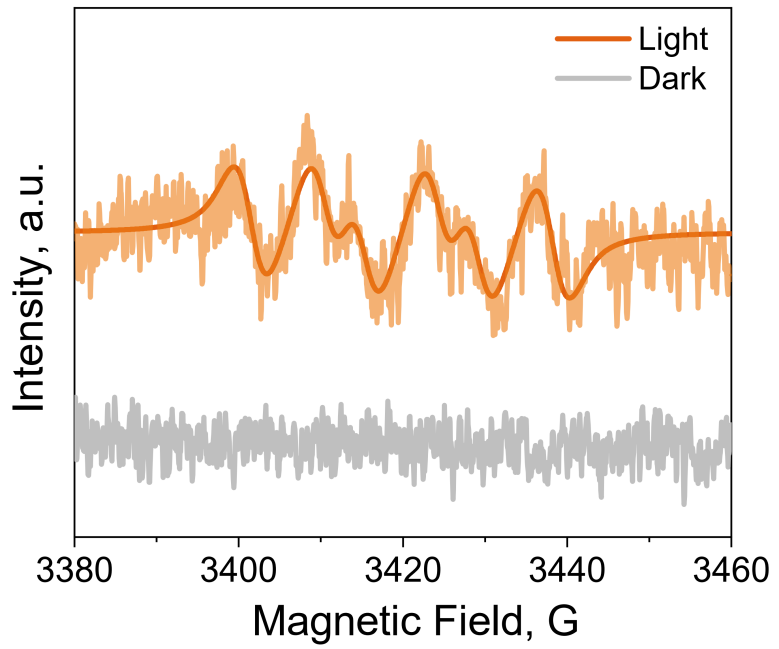


Fig. S5. EPR spectra of superoxide radicals.


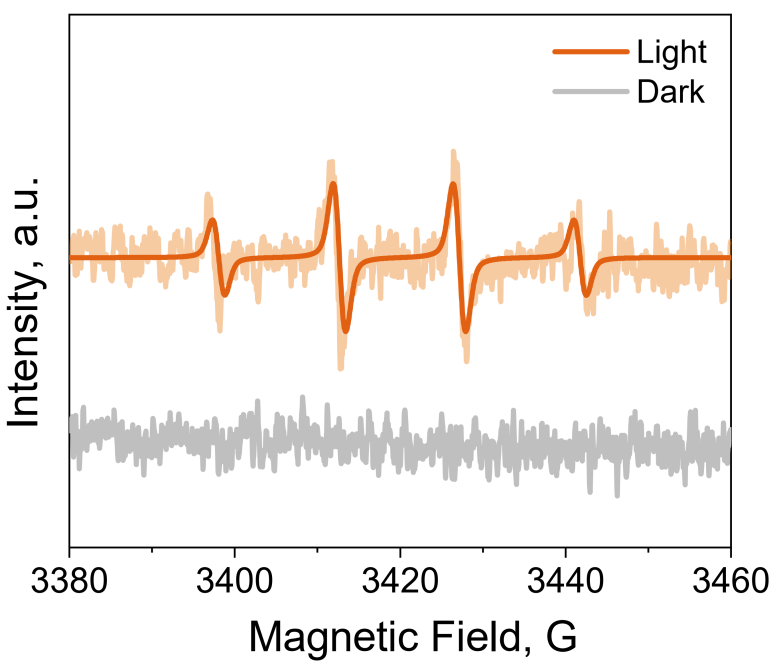


Fig. S6. EPR spectra of hydroxyl radicals.


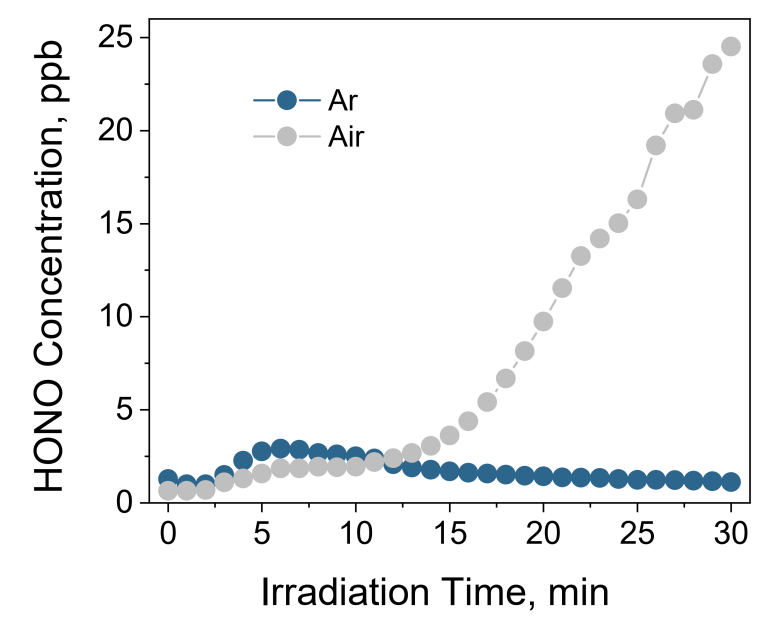


Fig. S7. HONO generation of NH_4_OH under irradiation in argon atmosphere at 75% RH.


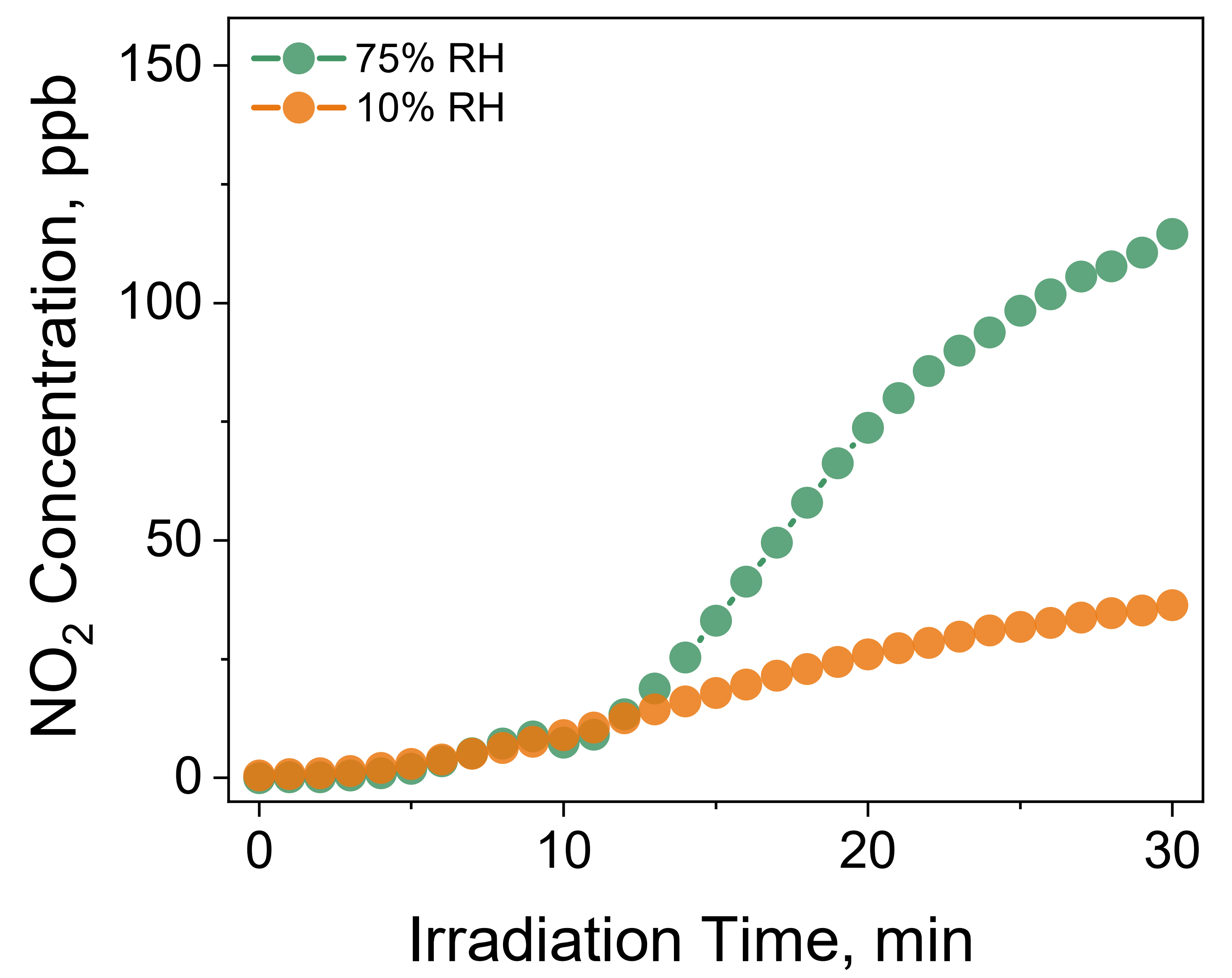


Fig. S8. NO_2_ generation of surface NaNO_3_ under light irradiation at 10% RH and 75% RH.


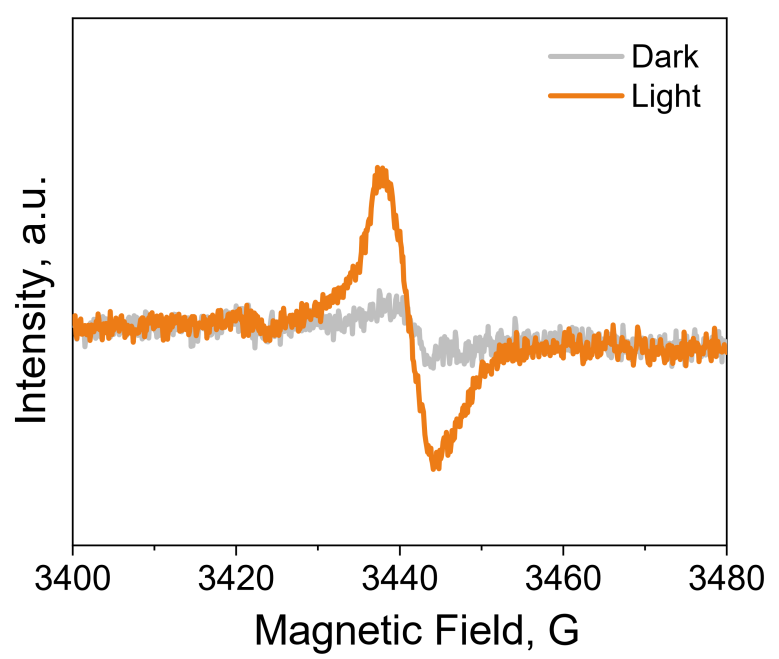


Fig. S9. EPR signal variation of TiO_2_ sample under irradiation.


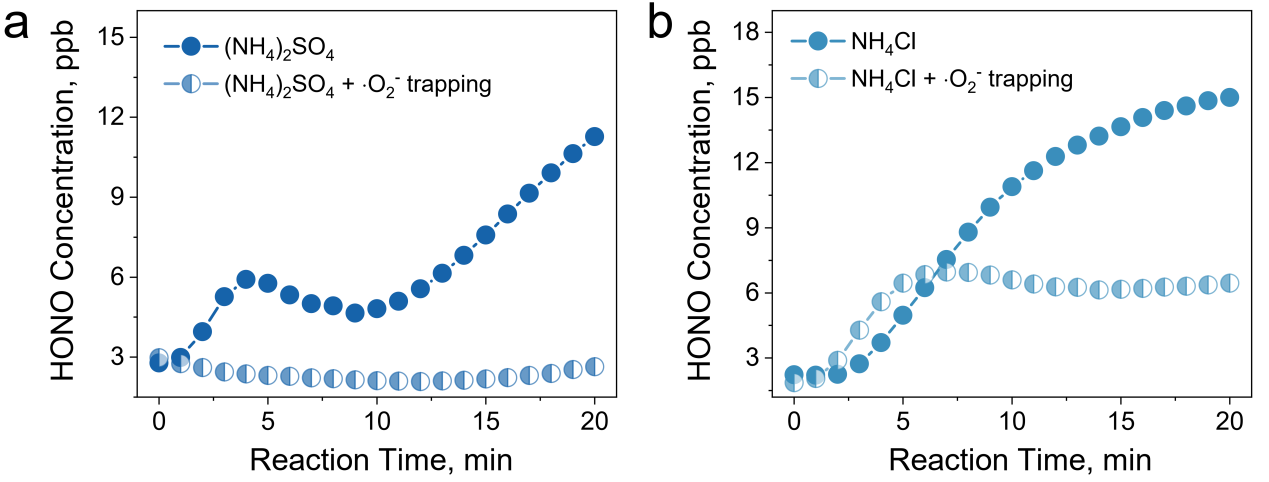


Fig. S10. HONO formation and ∙O_2_^-^ trapping of surface NH_4_SO_4_ (a) and NH_4_Cl (b) under simulated solar irradiation at high RH conditions.
